# Organizational Justice and Readiness for Change: A Concomitant Examination of the Mediating Role of Perceived Organizational Support and Identification

**DOI:** 10.3389/fpsyg.2018.01172

**Published:** 2018-07-10

**Authors:** Elodie Arnéguy, Marc Ohana, Florence Stinglhamber

**Affiliations:** ^1^Centre de Recherche et d’Études en Gestion, Université de Pau et des Pays de l’Adour, Pau, France; ^2^Psychological Sciences Research Institute, Université catholique de Louvain, Louvain-la-Neuve, Belgium; ^3^Kedge Business School, Talence, France

**Keywords:** readiness for change, overall justice, justice, perceived organizational support, organizational identification, organizational change

## Abstract

Survival in today’s global economy requires organizations to be flexible and adapt readily to the ever-changing marketplace. However, more than 70% of organizational change initiatives fail, mostly due to employees’ resistance to change. The literature has identified readiness for change (RFC) as an important cognitive precursor of resistance. A body of research has accordingly investigated the determinants of employees’ RFC. In particular, RFC has been shown to be positively predicted by employees’ perceptions of fair treatment. Little is known, however, on the mechanisms underlying this relationship. Relying on social exchange theory and social identity theory, this paper investigates the concomitant mediating role of perceived organizational support (POS) and organizational identification (OID) between overall justice and RFC. One hundred and forty-five employees of a company located in France participated in a survey-based study. Results of the path analyses indicated that POS mediates the positive effect of organizational justice on RFC, while OID does not act as a mediator in this relationship. As a whole, these results show the relevance of social exchange theory to better understand how employees become ready to change in organizational settings.

## Introduction

As a decisive factor for long-term success and survival of an organization, change has become an inherent and integral part of organizational life (By, 2005). However, about two-thirds of organizational efforts to implement planned change fail (Beer and Nohria, 2000; Meaney and Pung, 2008). Employees’ attitudes are a central cause for change projects failure (Choi, 2011). Extending knowledge about the psychological mechanisms of employees’ reactions to specific change initiatives is thus invaluable to both scholars and practitioners.

Readiness for change is recognized as a cognitive precursor of resistance to change and, conversely, of change support (Armenakis et al., 1993). It refers to employees’ beliefs, attitudes, and intentions regarding the extent to which changes are needed and the organization is capable to successfully implement those changes (Armenakis et al., 1993). Given the crucial role that RFC plays in organizational changes successes (Neves, 2009; Haffar et al., 2013; McKay et al., 2013; Santhidran et al., 2013), it has received a growing attention from scholars and is therefore currently viewed as a key change attitudinal variable (Choi, 2011). The present study aims at contributing to the examination of the antecedents of RFC by addressing three important research questions that have received relatively little attention.

First, we draw on Lind (2001) FHT to suggest that overall justice influences RFC. Although one study has showed that distributive and procedural justice affect RFC (Shah, 2011), further examination is required to examine the impact of overall justice on RFC. According to the FHT, employees develop an overall and initial fairness judgment that stays stable over time. This fairness evaluation is of importance since it influences employees’ reactions to subsequent events and guides their behaviors (Lind, 2001). Justice scholars have echoed such an argument by calling for more research on overall justice (Greenberg, 2001; Shapiro, 2001; Ambrose and Schminke, 2009). Yet, to date, limited empirical work has examined the effect of overall justice in the context of change (Rodell and Colquitt, 2009; Marzucco et al., 2014). To address this issue, we propose to examine the effect of overall justice on RFC.

Second, we propose a much-needed empirical investigation of the FHT underlying mechanisms, by investigating the impact of overall justice on RFC through two major theories, i.e., the social identity theory and the social exchange theory. As noted by Blader and Tyler (2005), the mechanisms explaining the justice-outcomes relationship within the FHT framework remain an open question. More precisely, this study addresses this important research question by simultaneously investigating the mediating role of POS and OID in the overall justice-RFC relationship. Substantial theoretical developments and empirical evidence argue for the decisive mediating role of these two variables. However, to the best of our knowledge, no study has considered the mediating role of POS in the justice-change responses relationship. Furthermore, research has shown mixed results concerning the mediating role of OID between justice dimensions and change-related variables (Michel et al., 2010; Fuchs and Edwards, 2012).

Third, the concomitant examination of these two central processes permits a closer examination of their relative effect, and thus advances prior empirical findings examining their concurrent contribution in the explanation of relationships (Cho and Treadway, 2011). Despite the key role played by social identity and social exchange processes in the development of prosocial reactions, these theories have been either studied separately (Olkkonen and Lipponen, 2006), sequentially (Edwards, 2009; Marique et al., 2013; Caesens et al., 2014; Stinglhamber et al., 2015) or treated interactively (Van Knippenberg et al., 2007; Tavares et al., 2016). The only exception is one recent study of Cho and Treadway (2011) who found that, when considering simultaneously the mediating role of POS and OID, OID mediates the relationship between procedural justice and organizational citizenship behavior whereas POS did not. The present study follows the same logic by proposing to examine the concomitant role of these two mediators treated in parallel. We assume that the two mechanisms they embody may complement each other instead of competing with one another.

In the following sections, we explain the theoretical processes of the model in more detail. Next, we describe the study design. Finally, we present the results and its implications for research and practice.

## Theoretical Background

### Justice and Readiness for Change

According to Lewin (1952) landmark field theory and three-step model, behavior is the product of the interaction of two types of forces: the driving forces, which push for change, and the restraining forces, which press in the opposite direction. When both forces are equal, the ongoing behavior is maintained at a quasi-stationary equilibrium. Behavioral change results from the alteration of these forces and should gradually proceed through the stages of unfreezing, moving and refreezing, in order to be successful.

As the opening step of the change process, unfreezing is a decisive stage for the development of change. Accordingly, it has been argued that change failures often result from an ineffective unfreezing process before moving to the other change steps (Schein, 1987, 1999; Kotter, 1995, 1996). Schein (1996) identified three forces prompting the individual to shift from the status quo to the unfreezing stage: the induction of guilt or survival anxiety, the disconfirmation of the validity of the status quo, and the creation of psychological safety. People will thus respond to change only if they perceive that quitting their current situation is unavoidable. They also need to be comforted to know that the foreseen change will not threat them and that the required means to achieve it are present. Otherwise, they will defend themselves by preserving the status quo.

As numerous researchers pointed out, RFC captures this critical stage by revealing if the altering forces of the unfreezing stage are in progress (Armenakis et al., 1993; Holt et al., 2007b; Choi and Ruona, 2011; Ford and Foster-Fishman, 2012; Vakola, 2013). In other words, it shows whether employees are successfully passing through the unfreezing stage and thus will be prepared to face the upcoming change. This progression is likely to be successful if employees perceive the relevance of change to the goal attainment (disconfirmation of the validity of the status quo) and the impasse of the company current situation (induction of guilt or survival anxiety). Employees will not resist these forces to the extent that they perceive no threat related to the change thanks to securing mechanisms signaling them that the change will turn out well (creation of psychological safety).

Given this decisive role of RFC in the change process, a growing number of conceptual and operational definitions has been suggested (Choi, 2011). Most of them tend, however, to shed light on similar dimensions (Armenakis et al., 2007; Holt et al., 2007b; Choi, 2011). Building both on an integrated conceptual framework and the development of a measure instrument, Holt et al. (2007a) have provided a definition and an operationalization of RFC which are certainly considered as one of the most comprehensive and robust in the readiness literature (Ford and Foster-Fishman, 2012; Stevens, 2013). More specifically, they proposed that RFC “reflects the extent to which an individual or individuals are cognitively and emotionally inclined to accept, embrace, and adopt a particular plan to purposefully alter the status quo” (Holt et al., 2007a, p. 235). As such, it is considered as “the cognitive precursor to the *behaviors of* either resistance to, or support for, a change effort” (Armenakis et al., 1993, p. 681).

Within this framework, RFC consists of four dimensions: (a) appropriateness; (b) management support perception; (c) change self-efficacy; (d) personal valence perception (Holt et al., 2007a). Appropriateness refers to the employee’s assessment of the change adequacy for resolving the issues faced by the organization. More specifically, when a specific change is introduced by their organization, individuals evaluate whether a change is required by estimating whether the current situation prevents their organization from reaching a more desirable state. The proposed change is considered as appropriate if the employee feels it corresponds to the right response to the situation faced by the organization. The second dimension, i.e., management support, refers to individual’s beliefs that key organizational members, such as top decision-makers and senior leaders, are fully supportive of the particular change and committed to the proposed change and its success by, for example, emphasizing its importance and encouraging employees to adopt it. Change self-efficacy is defined as employees’ perceptions that they are capable to implement the proposed change. It thus refers to their assurance that they will effectively cope with the prospective change by possessing the level of skills associated with the implementation of the change. Finally, personal valence refers to employees’ perception that the proposed change is beneficial to them. This latter dimension is thus related to the personal gains one may obtain from the successful implementation of the change. It stands for the “what is in it for me” question. For example, employees might assess whether the change will improve their status, their relationship and their future in the company.

Given the significance of RFC for the change success, it is no surprise that a growing body of empirical research has evidenced the impact of RFC on change success-related outcomes, including resistance (McKay et al., 2013) and employees’ level of individual change (Neves, 2009). Organizational change scholars have also explored the antecedents of RFC and showed that it is driven by change-related factors, such as communication adequacy and participation (McKay et al., 2013) and organizational factors, including distributive and procedural justice (Shah, 2011).

Organizational justice refers to employees’ perception of fair treatment by their organization. Researchers have traditionally considered that organizational justice is best represented by four distinct dimensions, namely distributive, procedural, interpersonal, and informational justice. Distributive justice refers to the perceived fairness of outcomes. Procedural justice concerns the perceived fairness of the allocation process which leads to outcome decisions. Interpersonal justice refers to the perceived fairness of the extent to which one is treated with dignity and respect and informational justice reflects the perceived fairness of the extent to which one is provided with adequate information for decisions (Colquitt, 2001). Although empirical evidence supports the multidimensionality of justice and demonstrates the relationship between each form of justice and a wide range of employees’ attitudes and behaviors (Colquitt et al., 2013), some scholars advocated that the dimensions of justice may not accurately represent the individual’s justice experience in the workplace. They thus suggested a shift in attention to a more holistic appreciation of the justice judgments (Greenberg, 2001; Shapiro, 2001). This overall judgment of justice is argued to ultimately drive behavior and thus to be the main central mechanism, compared to the dimensions of justice (Ambrose and Schminke, 2009). In their seminal article, Ambrose and Schminke (2009) provided a definition of overall justice along with its measure. Overall justice reflects a global evaluation of the fairness of the treatment received by an entity and it is based on personal experiences as well as those of other group members (Ambrose and Schminke, 2009). Accordingly, empirical evidence has showed that although individuals can differentiate between the types of justice when prompted, this “overall justice” is a more proximal predictor of outcomes compared to the dimensions of justice (Ambrose and Schminke, 2009).

In a related vein, Lind (2001) FHT posits that employees rely on a cognitive shortcut or heuristic, referring to “a global impression of fair treatment” (Lind and van den Bos, 2002, p. 196). Moreover FHT provides a valuable conceptual framework for explaining the part played by a perception of overall justice in guiding employees when facing workplace changes. A central tenet of FHT is that this justice perception is a key element for individuals in deciding whether or not to cooperate with organizational authorities, because it helps them to resolve a fundamental social dilemma: either they cooperate with the authorities at the risk of being exploited, either they do not and renounce any benefit that may arise from cooperation. This dilemma is particularly acute during uncertain times, such as organizational changes (Lind, 2001; Lind and van den Bos, 2002). Consequently, employees have to choose whether to cooperate or not, while they are unsure about what lies ahead. Hence, they will especially rely on their overall justice perception to guide their responses to the forthcoming change.

Not surprisingly, an extensive number of empirical studies have provided evidence for the role of justice in shaping individuals’ responses to change (Fuchs and Edwards, 2012; Mitchell et al., 2012), including RFC (Shah, 2011). However, to our knowledge, only two studies have examined the effect of fairness as a global perception in change setting (Rodell and Colquitt, 2009; Marzucco et al., 2014). In line with this growing evidence of the influence of justice on employees’ attitudes and behaviors toward change, one can reasonably assume that overall justice will be positively related to RFC. More importantly, research exploring the underlying mechanisms of this relationship is scarce (Michel et al., 2010; Fuchs and Edwards, 2012). We suggest to rely on two theories – i.e., social exchange and social identity theories – which have been evidenced as relevant frameworks to explain the effects of justice (Tyler and Blader, 2003; Kurtessis et al., 2015). Theoretically speaking, there are at least two reasons why social identity and social exchange processes should complement each other to foster RFC. First, the core assumption of the FHT is that people use justice perceptions in order to resolve a fundamental social dilemma, by helping them decide whether to cooperate with the entity who has authority over them. More specifically, this dilemma raises two major concerns: “One aspect of the fundamental social dilemma (…) reflects a tension between the material rewards of organizational life and the possibility of exploitation. The source of the concern is that, by allowing one’s own outcomes to depend on the actions and choices of others, we run the risk that those others will take more than they give (…). If one chooses to behave cooperatively, one would like some guarantee – or at least some expectation – that others will not exploit that cooperative behavior. The other aspect of the fundamental social dilemma is the concern about linking one’s identity in a relationship, role, or organization and the danger or rejection that can threaten that identity” (Lind, 2001, pp. 62–63). Thus, according to the FHT framework, social exchange and social identity should be the core mechanisms explaining the effect of justice on beneficial reactions toward the organization. Second, social identity theory and social exchange theory are based on distinct assumptions, yet complementary (Van Knippenberg et al., 2007; He et al., 2014). As noted by Van Knippenberg et al. (2007), “social exchange processes imply a relationship in which the individual and the organization are separate entities psychologically [while] identification implies that the individual and the organization are one” (p. 463). Indeed, social exchange theory suggests that relationships are driven by reciprocation, implying that the two exchange partners are distinct. On the contrary, by inferring that the individual can merge one’s self with the organization, the social identity theory depicts the mechanisms leading to this union. As a whole, it clearly appears that the two theoretical frameworks rely on very different processes which may complement each other instead of competing with one another. Accordingly, our objective in the present study will be to examine how these two mechanisms may play a concomitant role in the relationship between overall justice and RFC. Concretely, POS and OID were used in the present study to capture social exchange and social identity, respectively. Below, we argue why these two variables may both mediate the overall justice-RFC relationship.

### Perceived Organizational Support as a Mediator

Over the past decade, social exchange theory has emerged as an important theoretical framework to shed light on the effects of justice (Colquitt et al., 2013). Drawing on Gouldner (1960) and Blau (1964) seminal works, social exchange theory explains relationships through the lens of transaction. Exchanges generate a mutual sense of obligation between the two parties of the relationship, through the norm of reciprocity, which implies an equivalent amount exchanged by both sides (Gouldner, 1960). In contrast with economic exchange which is based on rather explicit appreciation of each party’s duties, social exchange entails more unspecified obligations and involves less tangible resources (Blau, 1964).

Previous research indicates that POS is a meaningful concept for capturing social exchange between workers and their organization. POS refers to employees’ general beliefs concerning the extent to which the organization values their contributions and cares about their well-being (Rhoades and Eisenberger, 2002; Eisenberger and Stinglhamber, 2011). More specifically, POS captures the obligatory dynamics at play in exchange relationships (Cropanzano and Byrne, 2000; Colquitt et al., 2013), because it expresses the perception of a beneficial treatment which, as suggested by the reciprocity norm (Gouldner, 1960), generates afterward a feeling of obligation toward the source of this beneficial treatment.

In this study, we argue that POS mediates the positive relationship between overall justice and RFC. Firstly, employees’ justice perceptions are likely to foster a sense of being supported by the organization. When people feel that the organization acts fairly, they interpret such fair actions as signals indicating that the organization cares about them and values them (Eisenberger et al., 1986). There is abundant evidence that justice enhances POS (Rhoades and Eisenberger, 2002; Colquitt et al., 2013; Kurtessis et al., 2015). However, these previous studies have focused on the relationships between specific justice dimensions and POS (Edwards, 2009). Given that overall justice represents a general perception of the fair treatment received from the organization, overall justice may also influence POS.

Secondly, literature on social exchange provides arguments for a relationship between POS and RFC. Organizational support theory (Eisenberger and Stinglhamber, 2011) suggests that once people feel supported, they feel indebted to their organization through the activation of the norm of reciprocity (Gouldner, 1960). They are then more inclined to help their organization to reach its goals (Eisenberger et al., 1986; Shore and Shore, 1995). Empirical research shows that POS indeed induces a felt obligation toward the organization (Kurtessis et al., 2015) and, finally, positive work attitudes and behaviors (Rhoades and Eisenberger, 2002; Eisenberger and Stinglhamber, 2011; Kurtessis et al., 2015).

In line with the above, several studies demonstrate that POS mediates the relationship between fairness and beneficial attitudes and behaviors (Masterson et al., 2000). Surprisingly, no study has considered the potential mediating effect of POS in the justice-beneficial outcomes relationship in organizational change context. A few studies have nevertheless examined the effect of POS during organizational change. POS was for example found to relate to the perception of organizational capability to handle a team-based change (Eby et al., 2000), intention to use a new IT system (Magni and Pennarola, 2008; Mitchell et al., 2012), the enjoyment when using a new IT system, acceptance and use of a new IT system (Mitchell et al., 2012).

Building on the above development of the mediating role of POS between fairness and positive organizational outcomes, it is thus reasonable to assume that when workers perceive fairness in the organization they will feel supported by this organization, which in turn fosters a felt obligation to repay the organization through positive attitudes toward change. Thus, we hypothesize:

Hypothesis 1: POS mediates the positive relationship between overall justice and RFC (appropriateness, management support, self-efficacy, personal valence).

### Organizational Identification as a Mediator

Another approach, based on social identity, could explain why people engage more easily in changing environment when they feel treated with justice. Social identity theory assumes that people derive their identity from both individual identity and social identity. The latter comes from the feeling of belonging to one or several social groups. More precisely, it reflects “that part of an individual’s self-concept which derives from his knowledge of his membership of a social group (or groups) together with the value and emotional significance attached to that membership” (Tajfel, 1978, p. 78). A central assumption of social identity theory is that identification, especially with a valued group, satisfies individuals’ needs for achieving and maintaining a positive self-esteem (Ashforth et al., 2008).

Organizational identification is defined as “the perception of oneness with or belongingness to an organization, where the individual *defines* him or herself in terms of the organization(s) in which he or she is a member” (Mael and Ashforth, 1992, p. 104). In fact, when people identify themselves with a group, a part of their identity is blended into the group identity, so that “OID aligns individual interests and behaviors with interests and behaviors that benefit organization” (Dutton et al., 1994, p. 256). A solid body of empirical studies shows the positive effects of identification on desirable attitudes and behaviors toward the organization, such as job involvement, in-role performance and extra-role behaviors (Riketta, 2005; Lee et al., 2015).

In this study, we argue that OID mediates the positive relationship between overall justice and RFC. Firstly, justice perceptions may contribute to the development of identification. Theoretical support for this argument comes from the group engagement model proposed by Tyler and Blader (2003). This model proposes that the perception of being treated fairly gives information to people about the nature of their relationship with the group. They feel that they are treated with respect, and that they can be proud of belonging to the group. Thus, these feelings contribute to identification with the group (Tyler and Blader, 2003). Empirical studies have confirmed the effect of both the specific dimensions of justice (Olkkonen and Lipponen, 2006; Blader and Tyler, 2009; Fuchs and Edwards, 2012) and overall justice (Patel et al., 2012) on OID.

Secondly, OID may contribute to RFC. The alignment mechanism between individual and organizational interests carried by OID may be especially important in the context of organizational change, since employees’ attitudes and behaviors are expected to evolve in a way that will help the organization to reach its new objectives (Rousseau, 1998). Moreover, employees can make sense of the organizational change by relying on the meanings provided by the organization they identify with (Ashforth et al., 2008). Two different streams of empirical research have addressed identification in the context of change. In the field of mergers and acquisitions, studies have notably focused on the intergroup processes (Edwards and Edwards, 2012). The present study refers to the other line of research that has examined the relationship between OID and employees’ responses toward organizational change. Previous research has evidenced the positive impact of OID on RFC (Drzensky et al., 2012; Hameed et al., 2013).

Building on the above, we expect that, during change, when people perceive that they are treated fairly by their organization, they form a feeling of membership with it. This bond leads them to align their interests with the organizational objectives and expectations, prompting them to develop a state of readiness to face the upcoming changes laid out by the organization. The mediating role of OID between justice and prosocial responses has been confirmed by empirical research (Olkkonen and Lipponen, 2006; Blader and Tyler, 2009). In change settings, OID was found to mediate the relationship between the dimensions of justice and commitment to change (Michel et al., 2010). Unexpectedly, however, research has shown mixed results concerning the OID mediation between the dimensions of justice and change-related behaviors. Michel et al. (2010) field study revealed that, before the implementation of changes, the effect of procedural justice on commitment to change and change-supporting behaviors is partially mediated by OID. Nevertheless longitudinal survey data, collected when changes were in progress, showed that, whereas the mediating role of OID between procedural justice and commitment to change was confirmed, OID did not act as a mediator of the relationship between procedural justice and change-supporting behaviors. In the same vein, Fuchs and Edwards (2012) evidenced that, in the context of changes already implemented, among the four forms of justice, only interpersonal justice contributed to OID, which in turn leads to pro-change behaviors.

Here, we argue that OID plays a mediating role between overall justice and RFC, because RFC captures the state of preparedness to address changes that are not implemented yet and that employees have thus not experienced. In line with the FHT, we suggest that people rely on overarching justice perceptions in order to evaluate whether investing their self in the organization is safe and consequently define a part of themselves as a part of the organization. This process of identification contributes to join their interests with the organizational ones and to give them confidence in their ability to handle the forthcoming change. Therefore, we posit:

Hypothesis 2: OID mediates the positive relationship between overall justice and RFC (appropriateness, management support, self-efficacy, personal valence).

**FIGURE 1:**
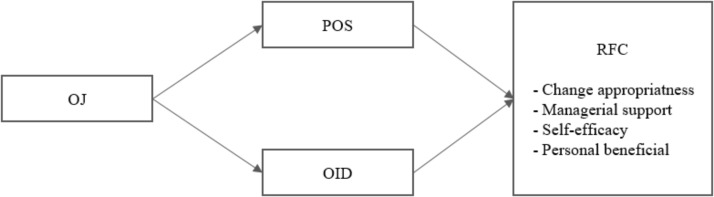
OJ, overall justice; POS, perceived organizational support; OID, organizational identification; RFC, readiness for change.

**Figure 1** presents the model of the study.

## Materials and Methods

Because RFC refers to the unfreezing stage, we choose to propose the study to a company that was about to implement a change and had not yet executed it. All in all, data were collected through surveys distributed in a French company based worldwide and operating in the energy industry. This company employs 150,000 people all over the world. The organizational change that was the focus of the present study concerned a specific branch exclusively based in France. This branch comprises 20,000 employees. At the time of the study, the firm had planned to implement a new information system involving cultural, organizational, and procedural changes in one of its industrial branch. Almost all the jobs were concerned by this change. Communication about the forthcoming change had been initiated. Given the complexity of the change, the company decided to sequentially implement it on a unit by unit basis, to cover all the units of the branch. At the time of the study, eight units were preparing the change.

We used two collection methods. Some questionnaires were distributed at the end of workshops about the change implemented in the company. Others were given by the change manager to people concerned by the change. In the latter case, a stamped envelope was supplied in order to ensure the anonymity of the respondents. In both cases, a cover letter explained the aim of the study. The survey contained the variables of interest and four control variables (i.e., age, organizational tenure, level of education, gender). To ensure that the participants would refer to the specific change described above, when answering to the change-related items, the name of the change project was used in these items.

Overall, 145 useable questionnaires were returned. As we do not have the number of employees approached by the change leader, the return rate is unknown. The respondents were in majority male (88%). This high proportion can be explained by the population of the energy industry where males are largely over-represented. Also, 42% had a master degree. This large proportion of highly educated respondents can be explained by the fact that the survey was addressed at the beginning of the communication about the project, while it was essentially high-qualified employees who were informed by the upcoming change. The average age was 42 years, with a range from 23 to 60 years (*SD* = 8.60). The average tenure was 12 years, with a range from a few months to 35 years (*SD* = 9.14).

### Measures

As the study was conducted in a French-speaking context, we relied on French versions of the scales used to measure all the variables included in our model. More precisely, the scales used to capture overall justice, POS and OID were already available in French from previous work (Marzucco et al., 2014; Stinglhamber et al., 2015). Regarding RFC, however, we translated the original scale in French using the translation-back-translation procedure recommended by Brislin (1980). Accordingly, the RFC measure was translated from English to French by a first bilingual Ph.D. student (unrelated to this research) and then independently back-translated by a professional translator. Afterward, the two translators assessed the minor discrepancies and made the necessary adjustments.

Except for the control variables, respondents were asked to answer on a 7-point Likert-type scale ranging from 1 (strongly disagree) to 7 (strongly agree).

*Overall justice* was assessed with the six-item scale developed by Ambrose and Schminke (2009). A sample item is 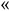 Overall, I’m treated fairly by my organization 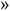. Cronbach’s alpha was 0.87.

*Perceived organizational support* was measured with the shortened eight-item version of the Survey of Perceived Organizational Support (Eisenberger et al., 1986). A sample item is “My organization really cares about my well-being.” Cronbach’s alpha was 0.87.

*Organizational identification* was measured with the six-item scale developed by Mael and Ashforth (1992). A sample item is “When someone criticizes my organization, it feels like a personal insult.” Cronbach’s alpha was 0.75.

*Readiness for change* was assessed with the 25-item scale developed by Holt et al. (2007a). As mentioned above, we substituted the word “change” by the name of the project impacting the employees, following Weiner (2009) recommendations to catch respondents’ attention to the specific change. Such adaptations of the change scales are frequently used in the change literature in general (Jiao and Zhao, 2014), and regarding the Holt et al. (2007a) RFC instrument in particular (Haffar et al., 2016). The scale has four dimensions: change appropriateness was measured with 10 items (e.g., “I think that the organization will benefit from the [name of the project].”); managerial support, measured with six items (e.g., “Our senior leaders have encouraged all of us to embrace the [name of the project].”); self-efficacy, measured with six items (e.g., “When we implement the [name of the project], I feel I can handle it with ease.”); personal valence, measured with three items (e.g., “My future in this job will be limited because of the [name of the project].”).

Internal consistencies of the four dimensions were acceptable: for appropriateness, Cronbach’s alpha was 0.86; for managerial support, Cronbach’s alpha was 0.86; for personal valence, Cronbach’s alpha was 0.80. Finally, for self-efficacy, Cronbach’s alpha was 0.73 after the deletion of one item (“I do not anticipate any problems adjusting to the work I will have when this change is adopted.”) weakly correlated with the other items of the scale.

### Control Variables

We measured four socio-demographic variables that have been shown to correlate with our variables of interest in prior studies. While both POS and OID have been found to be significantly related to age and organizational tenure (Riketta, 2005), POS is also associated with level of education and gender (Rhoades and Eisenberger, 2002). Accordingly, respondents were invited to specify their age, organizational tenure, level of education and gender. For level of education, the response scale was composed of six categories (middle school, senior high school, high school diploma, associate of arts, bachelor degree, and master degree and above).

As shown in **Table 1**, gender and tenure were not related to the dependent variables included in the study. Age displayed a significant correlation with OID (*r* = 0.23, *p* < 0.01), and level of education was significantly associated with POS (*r* = 0.18, *p* < 0.05), appropriateness (*r* = 0.20, *p* < 0.05) and personal valence (*r* = 0.20, *p* < 0.05). Therefore, following Becker (2005) recommendation, we finally controlled for age and level of education only in the subsequent analyses in order to reduce model complexity.

**Table 1:** Means, standard deviations, reliabilities, and correlations among variables.

Variable	*M*	*SD*	1	2	3	4	5	6	7	8	9	10	11
1. Age	42.01	8.57	-										
2. Gender	1.11	0.31	-0.12	-									
3. Tenure	11.60	9.14	0.47***	-0.11	-								
4. Level of education	3.73	1.29	-0.35***	0.16	-0.48***	-							
5. Overall justice	4.75	0.97	0.06	0.07	0.10	0.15	(0.87)						
6. POS	4.38	0.94	0.02	0.06	0.03	0.18*	0.77***	(0.87)					
7. OID	5.28	0.77	0.23**	-0.03	0.12	0.10	0.40***	0.35***	(0.75)				
8. RFCAP	5.27	0.83	-0.14	0.14	0.07	0.20*	0.29***	0.35***	0.21*	(0.86)			
9. RFCMS	4.87	1.02	.09	-0.02	0.04	0.00	0.32***	0.36***	0.24**	0.36***	(0.86)		
10. RFCSE	4.48	0.88	0.13	-0.02	0.16	-0.03	0.32***	0.35***	0.17*	0.50***	0.31***	(0.73)	
11. RFCVAL	5.62	1.20	-0.05	0.11	0.00	0.20*	0.34***	0.40***	0.21**	0.47***	0.22**	0.43***	(0.80)

*OJ, overall justice; POS, perceived organizational support; OID, organizational identification; RFCAP, appropriateness; RFCMS, management support; RFCSE, self-efficacy; RFCVAL, personal valence; Gender (1, male, 2, female); Level of education is coded from 0 to 5. Scale reliabilities are reported in parentheses. ^∗^p < 0.05, ^∗∗^p < 0.01, ^∗∗∗^p < 0.001.*

## Results

Given the small size of the sample and the number of parameters to be estimated, we conducted two separate confirmatory factor analyses (CFA) in order to confirm the factor structure of the variables included in our model. The first one was performed on the antecedents of RFC (namely overall justice, POS and OID) since theoretical and empirical literature suggest their close association. Furthermore, the correlations among these variables were high in the present study, ranging from 0.35 to 0.77 (cf. **Table 1**). Results show that the hypothesized three-factor model (model 1) has the most adequate fit (**Table 2**), confirming that overall justice, POS and OID are distinct construct.

**Table 2:** Goodness-of-fit summary for confirmatory factor analyses of overall justice, POS and OID.

Model	*df*	χ^2^	RMSEA	CFI	SRMR	Δχ^2^ (vs. hypothesized model)
*Model 1*: Hypothesized three-factor model	167	322.688	0.08	0.89	0.07	–
*Model 2*: Two-factor model (combining overall justice and OID)	169	418.163	0.10	0.82	0.09	95.475^∗∗∗^
*Model 3*: Two-factor model (combining POS and OID)	169	426.561	0.10	0.81	0.09	103.873^∗∗∗^
*Model 4*: Two-factor model (combining POS and overall justice)	169	359.728	0.09	0.86	0.07	37.04^∗∗∗^
*Model 5*: One-factor model (combining the three constructs)	170	457.754	0.11	0.79	0.09	135.066^∗∗∗^

*N = 145. POS, perceived organizational support; OID, organizational identification; df, degree of freedom; χ^2^, minimum fit function chi-square; RMSEA, Root-Mean-Square Error of Approximation; CFI, Comparative Fit Index; SRMR, Standardized Root Mean Square Residual. ^∗^p < 0.05, ^∗∗^p < 0.01, ^∗∗∗^p < 0.001.*

Next, we did a CFA to confirm the relevance of the four-dimension model for the RFC construct. The model differentiating the four dimensions (model 1) has the best indices (**Table 3**).

**Table 3:** Goodness-of-fit summary for confirmatory factor analyses of RFC dimensions.

Model	*df*	χ^2^	RMSEA	CFI	SRMR	Δχ^2^ (vs. hypothesized model)
*Model 1*: Hypothesized four-factor model	246	482.613	0.08	0.84	0.09	–
*Model 2*: Three-factor model (combining appropriateness and personal valence)	249	578.001	0.10	0.78	0.10	95.388^∗∗∗^
*Model 3*: Three-factor model (combining management support and self-efficacy)	249	636.675	0.10	0.74	0.14	154.062^∗∗∗^
*Model 4:* Three-factor model (combining self-efficacy and personal valence)	249	550.496	0.09	0.79	0.09	67.883^∗∗∗^
*Model 5*: Three-factor model (combining management support and personal valence)	249	646.043	0.10	0.73	0.13	163.43^∗∗∗^
*Model 6*: Three-factor model (combining appropriateness and self-efficacy)	249	563.409	0.09	0.79	0.10	80.796^∗∗∗^
*Model 7*: Two-factor model (combining appropriateness, self-efficacy and personal valence)	251	644.260	0.10	0.73	0.10	161.647^∗∗∗^
*Model 8*: One-factor model (all four dimensions)	252	931.880	0.14	0.54	0.13	449.267^∗∗∗^

*N = 145. df, degree of freedom; χ^2^, minimum fit function chi-square; RMSEA, Root-Mean-Square Error of Approximation; CFI, Comparative Fit Index; SRMR, Standardized Root Mean Square Residual. ^∗^p < 0.05, ^∗∗^p < 0.01, ^∗∗∗^p < 0.001.*

Means, standard deviations, reliabilities, and Pearson correlations among variables are presented in **Table 1**. Results indicate that overall justice significantly relates with each RFC dimension, namely appropriateness (*r* = 0.29, *p* < 0.001), managerial support (*r* = 0.32, *p* < 0.001), self-efficacy (*r* = 0.32, *p* < 0.001), and personal valence (*r* = 0.34, *p* < 0.001). In line with our Hypotheses 1 and 2, overall justice is also significantly related to POS and OID (*r* = 0.77, *p* < 0.001 and *r* = 0.40, *p* < 0.001), which are both positively associated with all the RFC dimensions (significant *r*s ranging from 0.35 to 0.40 for POS and from 0.17 to 0.24 for OID). This is consistent with meta-analytic results showing the relationship between justice and POS (Colquitt et al., 2013; Kurtessis et al., 2015), and POS and OID (Kurtessis et al., 2015).

**FIGURE 2:**
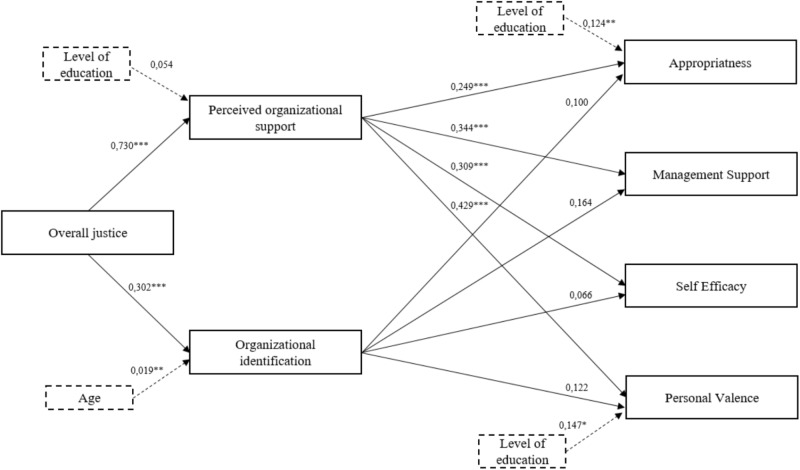
Dotted lines are used to represent the effects of control variables. (*N* = 145).χ^2^_(13)_ = 15.42, *p* = 0.28; RMSEA = 0.04; CFI = 0.99: CFI = 0.96; SRMR = 0.037. ^∗^*p* < 0.05, ^∗∗^*p* < 0.01, and ^∗∗∗^*p* < 0.001.

We then conducted path analyses using Lisrel 9.2 (Jöreskog and Sörbom, 2006) to examine the direct and indirect relationships hypothesized in the theoretical model (cf. **Figure 1**). The fit indices of the model showed an adequate fit to the data [χ^2^_(13)_ = 15.42, *p* = 0.28; RMSEA = 0.04; CFI = 0.99; CFI = 0.96; SRMR = 0.037]. Standardized parameter estimates for the model are presented in **Figure 2**. The results indicate that overall justice is positively associated with POS (β = 0.730, *p* < 0.001) which, in turn, is positively related to the RFC dimensions: appropriateness (β = 0.249, *p* < 0.001), managerial support (β = 0.344, *p* < 0.001), self-efficacy (β = 0.309, *p* < 0.001), and personal valence (β = 0.429, *p* < 0.001). Overall justice is also positively associated with OID (β = 0.302, *p* < 0.001). However, OID is not related to appropriateness (β = 0.100, *ns*), managerial support (β = 0.164, *ns*), self-efficacy (β = 0.066, *ns*) and personal valence (β = 0.122, *ns*). Furthermore, the indirect effects of overall justice on each RFC dimension were significant (appropriateness: 0.212, *p* < 0.001; managerial support: 0.301, *p* < 0.001; self-efficacy: 0.246, *p* < 0.001; and personal valence: 0.350, *p* < 0.001). As a whole, these results show that the effects of overall justice on RFC dimensions are mediated by POS only.

Finally, an alternative path model where overall justice was also allowed to have direct effects on the RFC dimensions was tested. This analysis indicates that the direct paths between overall justice and the RFC dimensions were not significant (appropriateness: 0.023, *ns*; managerial support: 0.060, *ns*; self-efficacy: 0.108, *ns*; and personal valence: 0.077, *ns*), suggesting that the effect of overall justice on RFC is fully mediated by POS. All together, these findings provide evidence for Hypothesis 1 but not for Hypothesis 2.

## Discussion, Limitations, and Managerial Implications

The aim of this study was to examine the relationship between justice and RFC, taking into account the potential concomitant mediation of both POS and OID. As evidenced by the results, the justice-RFC relationship is mediated by POS, while OID does not act as a mediator in this relationship. This research provides important insights for both theory and practice.

First, this study extends current knowledge on the determinants of RFC. Understanding its antecedents is crucial, since about two-thirds of change projects fail (Beer and Nohria, 2000; Meaney and Pung, 2008), mostly because of employees’ reactions to these projects (Choi, 2011). The results of this research confirm prior research (Shah, 2011) by providing evidence that justice indirectly contributes to the development of RFC. Additionally, they are consistent with the FHT (Lind, 2001), which suggests that overall justice perceptions help employees to decide about their work attitudes and behaviors especially in uncertain environments like organizational changes (Lind, 2001; Lind and van den Bos, 2002).

In addition, this study extends previous empirical findings by pointing out the key role of POS during organizational change. Precisely, the findings indicate that RFC derives from POS, which is in line with previous studies that examined the effects of POS on change-related attitudes (Eby et al., 2000; Magni and Pennarola, 2008; Mitchell et al., 2012). Moreover, there is no study testing the mediation of POS in the relationship between justice and change-related attitudes and behaviors. The present study filled this gap, by showing that social exchange derives from justice when there is an ongoing organizational change and fosters a state of readiness to change. This finding is important, since it suggests an additional explanatory mechanism within the FHT framework.

Another contribution of this study is its focus on OID. Contrary to the prediction, OID does not act as a mediator in the overall justice-RFC relationship. Nonetheless, as previously noted, OID is correlated with overall justice and the four dimensions of RFC. This is consistent with prior theoretical and empirical research, pointing out the role of justice in developing OID on the one hand (Tyler and Blader, 2003), and the impact of OID on RFC on the other hand (Drzensky et al., 2012; Hameed et al., 2013). Despite these links, OID does not mediate the effect of overall justice on RFC (at the very least in presence of POS, as discussed below). Thus, the identification with the organization deriving from a global fairness perception does not produce the initial shift toward change. In a similar vein, previous research on the mediation of OID between justice dimensions and change-related behaviors has showed controversial results (Michel et al., 2010; Fuchs and Edwards, 2012). More research is necessary to determine the generalizability of these findings and/or to identify the potential boundary conditions of these relationships.

Next, to the best of our knowledge, this research is the first one to integrate the social exchange and social identity theories (through the inclusion of POS and OID, respectively) in the study of the effect of justice on change-related attitudes. Our findings indicate that POS mediates the overall justice-RFC relationship, whereas OID does not. These results are important since they suggest that overall justice influences change attitudinal variables through social exchange dynamics. Furthermore, they add to the findings of recent research examining whether social exchange and social identity act as complementary or competing mechanisms (Cho and Treadway, 2011).

It clearly appears that, in changing environments, only a social exchange process explains the effect of justice on preparedness to change. At this stage, we can only make assumptions on the reason why POS acted as a mediator, whereas OID did not.

One possible explanation for this unexpected finding is that employees who are highly identified with their organization feel the forthcoming change is a threat to their current organizational identity. Employees might believe that the proposed change will lead to the alteration of the organizational attributes on which their OID is based. Accordingly, OID would not contribute to the acceptance of the organizational change. Similar arguments emerge from the OID literature (Drzensky and van Dick, 2013; Hameed et al., 2013), which stresses that individuals may feel their organizational identity is threatened when their organization is being transformed. On the contrary, employees may consider that changes will not jeopardize the treatment they receive from the organization since high-quality support is a strong indicator of organizational sincerity and benevolence (Eisenberger and Stinglhamber, 2011). Employees may believe that the favorable treatment provided by the organization is durable and preserved from any fundamental change in the HR policies and practices of their organization, because of its genuineness. Thus, while change might endanger individuals’ identification, it may not threaten the employees’ confidence in the continuity of the treatment brought by their organization. That would explain why OID does not predict anymore RFC as soon as POS is controlled for. Clearly this explanation is *post hoc* and would need further examination in future research.

Another possible explanation for the divergent role of POS and OID may be related to the stage of the change process of this study and the change-related constructs of interest. As pointed out earlier, the previous contradictory results about the mediating effect of OID in change settings might be explained by the change stage and the change-related variables under examination (Michel et al., 2010; Fuchs and Edwards, 2012). Although OID did not mediate procedural justice and commitment to change during the change process, OID acted as a mediator of the procedural justice-pro-change behaviors relationship 6 months after (Michel et al., 2010). Thus, the stage of change process or the change-related variables or both might explain these divergent findings. An interesting avenue for future research would be comparing both the stage of change and the change-related constructs. For example, although RFC is considered to refer to the unfreezing stage (Armenakis et al., 1993), some authors have suggested that RFC may also refer to other stages of the change process (Stevens, 2013). In a related vein, some researchers, such as Stevens (2013), have argued that RFC evolves through all the change process. Consequently, it might be expected that the RFC dimensions unfold differently, in particular given their distinctive feature. As another example, theoretical arguments suggest that the change-related concepts (e.g., RFC, commitment to change, pro-change behaviors) should sequentially be at stake throughout the change, as the change implementation implies that behavioral components gradually prevail over cognitive and affective dimensions (Stevens, 2013). For all these reasons, conducting research on employees’ reactions along the whole change process might certainly constitute a promising direction for future studies.

Importantly, based on the fact that POS and OID rely on different mechanisms, our objective in the present study was to test their concomitant role in the relationship between overall justice and RFC. Accordingly, we treated POS and OID as two mediators in parallel in this relationship. By doing so, we did not acknowledge the link or the interaction that may exist between the two constructs. Prior studies have indeed shown that perceiving support from one’s organization may lead the employee to identify to it (Edwards, 2009; Marique et al., 2013; Caesens et al., 2014; Stinglhamber et al., 2015). In another vein, several authors have found that the relationship between POS and outcomes is strengthened or, on the contrary, attenuated by a high level of OID (Van Knippenberg et al., 2007; Tavares et al., 2016). Our approach is complementary to the sequential examination of these constructs and the study of their interactive effects. Overall, these different research directions in the POS–OID relationship simply respond to different research questions. As a whole, we believe these lines of research are promising in extending our understanding of both the social identity theory and the social exchange theory and their interplay.

This study has also its own limitations. First, the sample is limited and had been collected in a single organization based in France. Only one sector (energy industry) is represented. Although this method of data collection minimizes the likelihood of unmeasured influences imputable to differences in organizations, the model deserves to be replicated in order to generalize these findings. Second, we rely in this study on self-reported data which expose our results to the common method bias (Podsakoff et al., 2003). Nevertheless, the results of the Harman single-factor test that we performed indicated a very poor fit of a one-factor model (cf. **Tables 2**, **3**). This evidence thus reduces our concerns regarding this threat. Third, because the results of our study are based on a cross-sectional design, the causal relationship among the variables is not demonstrated, as a longitudinal design with repeated measures would do. Finally, our hypotheses do not suggest that our mediating variables may have distinct effects on the different dimensions of RFC. Further, our findings show that each of RFC dimensions is significantly related to the same antecedent, namely POS, whereas OID relates poorly to each of the four dimensions. Yet, it might be considered, at first sight, that the RFC four dimensions – change appropriateness, managerial support, self-efficacy and personal benefits (Holt et al., 2007a) – are quite distinct, since some of them might be more oriented to organizational factors (i.e., change appropriateness and managerial support) and others to individual issues (i.e., self-efficacy and personal benefits). Future research proposing theoretical models that differentiate antecedents and outcomes for each of the sub-dimensions may lead to promising results that will help to better understand the change phenomenon.

From a practical standpoint, the present paper suggests that general organizational factors, as overall justice and POS, are relevant for people to be ready to change. It is thus important for management to care about these factors, regardless there is an ongoing change. In fact, if management aims at facing change stakes, they should foresee these challenges well in advance, by increasing these two organizational factors in the long term. Many types of actions foster POS, such as high-quality communication, job security, training and developmental opportunities (Eisenberger and Stinglhamber, 2011). The effect of these actions are all the more important, that they are viewed as discretionary by employees (Eisenberger and Stinglhamber, 2011). Furthermore, because justice is a crucial antecedent of POS, organizations should pay special attention to developing fairness by enhancing the recruitment process fairness (Gilliland and Hale, 2005), managing stress (Vermunt and Steensma, 2005) and reducing discrimination (Stone-Romero and Stone, 2005). Managers could benefit from being trained to conduct fairly these actions (Skarlicki and Latham, 2005). Given the increasing pace of organizational changes within the companies, it is crucial that management provides fair treatment and support to employees.

## Data Availability Statements

The raw data supporting the conclusions of this manuscript will be made available by the authors, without undue reservation, to any qualified researcher.

## Ethics Statement

This study was carried out in accordance with the recommendations of the APA Ethical Principles of Psychologists and Code of Conduct with written informed consent from all subjects. All subjects gave written informed consent in accordance with the Declaration of Helsinki. The protocol was approved by the ethics committee of the Psychological Sciences Research Institute of Université catholique de Louvain (UCL) in Belgium.

## Author Contributions

EA, MO, and FS contributed conception and design of the study. EA collected the data and organized the database. EA and MO performed the statistical analysis. EA wrote the first draft of the manuscript. EA, MO, and FS wrote subsequent versions of the manuscript. All authors contributed to manuscript revision, read and approved the submitted version.

## Conflict of Interest Statement

The authors declare that the research was conducted in the absence of any commercial or financial relationships that could be construed as a potential conflict of interest.

## Abbreviations

Fht: fairness heuristic theory
Oid: organizational identification
Pos: perceived organizational support
Rfc: readiness for change
